# 68 The Association Between Body Mass Index and Physical Function in Adult Burn Survivors

**DOI:** 10.1093/jbcr/irac012.071

**Published:** 2022-03-23

**Authors:** Alen Palackic, Victoria G Rontoyanni, Ludwik K Branski, Robert P Duggan, Jeffrey C Schneider, Colleen M Ryan, Karen J Kowalske, Nicole S Gibran, Barclay T Stewart, Steven E Wolf, Oscar E Suman-Vejas, David Herndon

**Affiliations:** University of Texas Medical Branch, Galveston, Texas; University of Texas Medical Branch, Galveston, Texas; University of Texas Medical Branch, Galveston, Texas; University of Texas Medical Branch, Galveston, Texas; , Massachusetts; Harvard Medical School, Boston, Massachusetts; Department of Surgery, The University of Texas Southwestern Medical Center, Dallas- Fort worth, Texas; University of Washington, Wellfleet, Massachusetts; University of Washington, Seattle, Washington; University of Texas Medical Branch at Galveston, Galveston, Texas; U of Texas Medical Branch, Galveston, Texas; UMTB, Galveston, Texas

## Abstract

**Introduction:**

An area of rehabilitation research in burns is the impact of co-morbidities. Obesity is one of these, is an increasing public health concern, and its role remains controversial regarding burn injury and physical recovery. Our aim was to evaluate associations between body mass index (BMI) as a measure of obesity, at discharge and self-reported physical function (PF) during recovery of adult burn survivors.

**Methods:**

This study included data that was collected by four American Burn Association-verified burn centers, which contribute to the Burn Model System National Database project. The data included BMI obtained at hospital discharge and self-reported Patient-Reported Outcomes Measurement Information System (PROMIS)-29 PF-mobility and upper extremity scores assessed at 6-, 12-, and 24-months after burn. Mixed linear models for repeated measures and regression models were used to assess associations between BMI and PROMIS-29 PF scores over time. Values are expressed as means ± SD. Significance was set at p< 0.05.

**Results:**

A total of 502 adult patients aged 47 ± 16 years were included, with mean total body surface area burned (TBSA) of 17 ± 18 % (range; 1.0-88%) and mean BMI of 23.1 ± 5.4 kg*m^-2^ (range; 14.0-64.7 kg*m^-2^). We found no significant effect at 6 months (beta=-0.045, p= 0.54) nor at 12 months after injury (beta=-0.063, p= 0.44) when adjusted for age, burn size, and sex, however, BMI at discharge had a significant negative effect on self-reported mobility scores 24 months after injury (beta=0.218, p=< 0.05).

**Conclusions:**

Increased weight (i.e. BMI) at discharge was negatively associated with PF during recovery. Benefiting from a large sample size, our analysis suggests that long term recovery and restoration of PF in adult burn survivors is compromized by excess body weight.

